# Novel 3D Liquid Cell Culture Method for Anchorage-independent Cell Growth, Cell Imaging and Automated Drug Screening

**DOI:** 10.1038/s41598-018-21950-5

**Published:** 2018-02-26

**Authors:** Natsuki Abe-Fukasawa, Keiichiro Otsuka, Ayako Aihara, Nobue Itasaki, Taito Nishino

**Affiliations:** 1 Biological Research Laboratories, Nissan Chemical Industries LTD, Saitama, Japan; 2 Faculty of Health Sciences, University of Bristol, Bristol, United Kingdom

## Abstract

Cells grown in three-dimensional (3D) cultures are more likely to have native cell-cell and cell-matrix interactions than in 2D cultures that impose mechanical constraints to cells. However, most 3D cultures utilise gel matrix which, while serving as a scaffold, limits application due to its solid and opaque nature and inconsistency in cell exposure to exogenous signals. In 3D culture without gel matrix, cells tend to adhere to each other and form clumps with necrotic zone at the centre, making them unsuitable for analyses. Here we report that addition of low-molecular-weight agar named LA717 to culture media allows cells to grow as dispersed clonal spheroids in 3D. LA717 maintains cells dispersed and settled to the bottom of the medium while keeping the medium clear with little additional viscosity, making it suitable for microscopic observation. Importantly, cancer spheroids formed in LA717-containing medium show higher sensitivity to anti-cancer drugs such as Trametinib and MK-2206 that are not as effective in 2D. Because of the small and consistent size of spheroids, cell viability and drug toxicity are readily detectable in automated imaging analysis. These results demonstrate that LA717 offers a novel 3D culture system with great *in vivo* reflection and practicality.

## Introduction

Cells in the physiological microenvironment undergo complex cell-cell and cell- extracellular matrix (ECM) interactions as well as being exposed to extracellular signals, all of which are the basis of multicellular organisms. Nonetheless, most cell-based *in vitro* studies conventionally employ two-dimensional (2D) monolayer cell culture which does not fully reflect cyto-architecture, cell interaction and response to exogenous stimuli. For example, large scale drug-screening often employs 2D cultures for practicality, but this may mask active compounds or select false-positives that turn out to be ineffective in *in vivo* studies^1,2^. As such, *in vitro* cell culture methods with high reliability to predict *in vivo* activities are desirable for the successful drug screening as well as for other cell studies.

Current 3D culture methods involve either the use of scaffolds such as gel matrix and micro-carriers, or liquid cultures on low-attachment plates, in hanging drops or in rotation^3,4^. While they have many advantages compared to 2D cultures, they also have practical challenges. For example, commercial gel matrices derived from basal lamina of osteosarcoma (e.g. Matrigel®) serve as a scaffold and maintain epithelial features very well; however, application of drugs is often hindered by the semi-solid matrix, making it unsuitable for high throughput screening (HTS). The temperature-dependent solidification and high opacity are also disadvantageous for automated liquid handling and imaging^3–6^. Liquid culture without gel matrix on low-attachment plates, on the other hand, can be handled as a clear liquid thus offering practicality. However, cell aggregates (spheres or spheroids) generated in the medium often form large clumps of >500 μm diameter due to cell adhesion, which causes a slow cell proliferation rate and poor diffusion of nutrients. Such generated large-sized spheroids may lead to pseudo-resistance to anti-cancer drugs^5^. Hanging drop method generates a uniform size of spheroid but requires considerable effort and special apparatus^4,6^. Microfluidic technologies provide a variety of practical platforms for 3D cell cultures and cell-based assays. However, these technologies remain relatively expensive owing to the microfabricated instruments required and the complexity of the process^7,8^. Furthermore, current cell-based HTS employs automated imaging system which allows analysis of cells with simultaneous data collection on several parameters, so-called high content imaging analysis (HCA) or high content screening (HCS) technology^9–11^. Several studies have examined implementation of 3D cultures in HCA/HCS using Matrigel, methyl cellulose or special micro-patterned plates to form spheroids^12–16^. However, a more practical and efficient method is desired to perform HCA/HCS in 3D cultures^17^.

In this study, we explored a novel 3D cell culture method by screening natural polysaccharides that would promote uniform suspension of cells on low-attachment culture plates while maintaining the practicality for liquid handling. We have identified low molecular weight agar (LA) polymers as suitable additives for the 3D cell culture platform and named it LA717. By lowering the molecular weight of agar polymers, it has become possible to increase the solubility of agar to the medium. We found that LA717 holds cells in the medium, keeping movement at a minimum and thus maintaining the even distribution of cells. Moreover, such well-distributed cells rarely make new contacts to others, hence, individual spheroids are largely maintained as clones. Although, cell adhesions following cell division or natural contacts are not affected by LA717. Lastly, we demonstrate that spheroids formed in the LA717-containing medium effectively reveal the action of anti-cancer drugs and are thus suitable to HCS, offering a novel 3D cell culture system which elicits more efficient and practical HCS systems.

## Results

### Selection and characterization of low molecular weight agar LA717 for 3D cultures

Polysaccharides are carbohydrates consisting of sugar molecules bound as polymers. The examples include agar, agarose and methyl cellulose, that have been used traditionally for biological studies such as colony-forming assays. We examined eight polysaccharides at different concentrations on lung cancer cells A549 for effective cell dispersion and uniform spheroid formation on low-attachment plates (Supplementary Table S1). With regards to agar^18–20^, which is a mix of agarose and agaropectin, we tested two types, one with a low average molecular weight (43,000)^21,22^ and a standard (290,000) one. Agar with standard molecular weight and other polysaccharides formed variable sizes of aggregates at certain concentrations in the medium due to the low solubility to DMEM (Supplement Table S1). Even when high solubility was achieved with some polysaccharides, many of them did not cause cell dispersion (Supplement Table S1). In some conditions, cells were successfully dispersed in the polysaccharides, however, cells were suspended in the medium without falling to the bottom of the plate. This is not a problem as a 3D culture, however, it is not ideal for imaging analyses and was hence eliminated (Supplementary Table S1). We found low molecular weight agar to be the most satisfactory polysaccharide for the above purpose at a concentration of 0.03% (w/v) and named it LA717.

**Figure 1:**
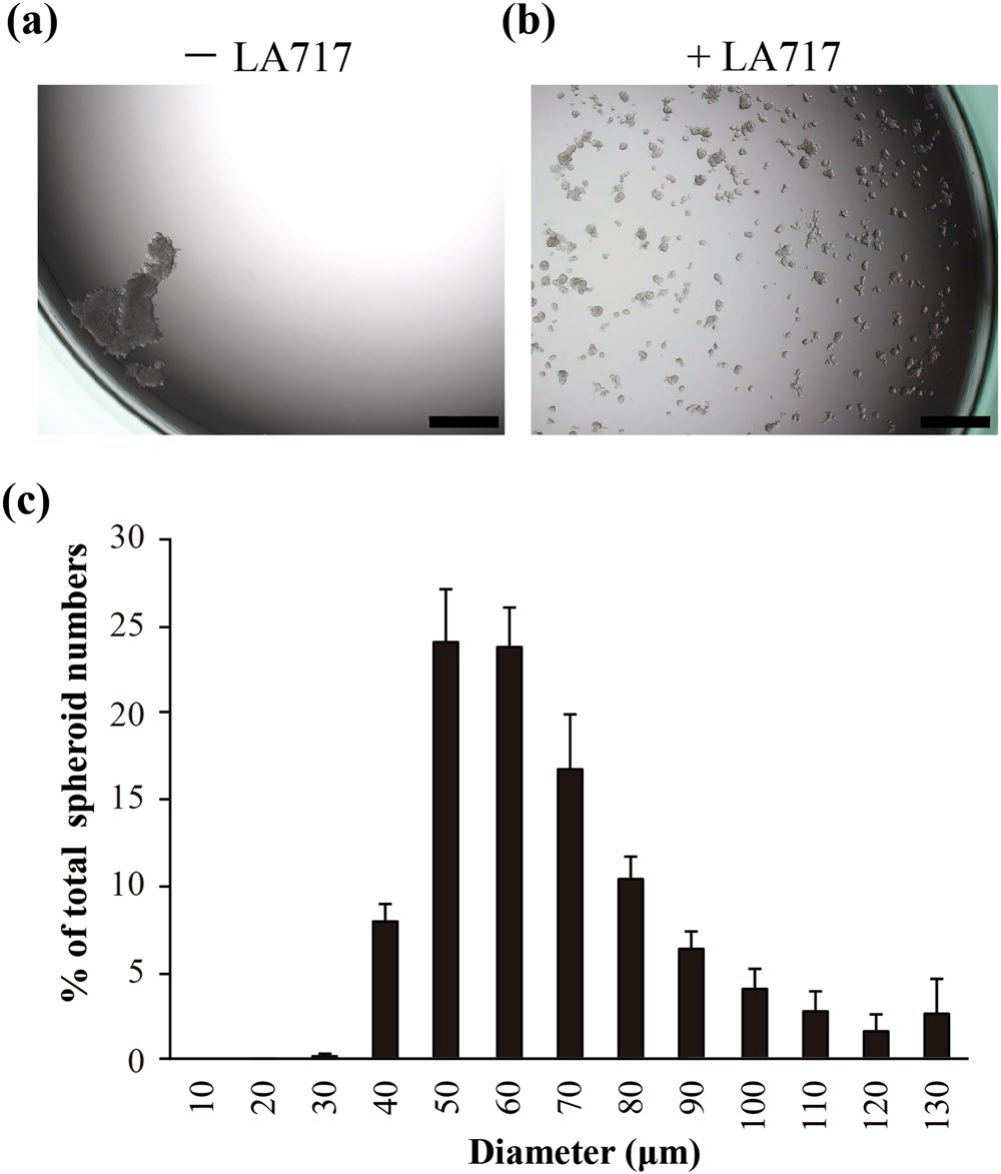
Identification of low-molecular weight agar (LA717) as a substrate for 3D cell culture. (**a**,**b**) A549 cells cultured for 7 days in the wells of low-attachment plates in the medium without (**a**) or with 0.030% (w/v) LA717 (**b**). Scale bar, 500 μm. (**c**) Size distribution of A549 spheroids formed on low-attachment plates in DMEM with 0.030% (w/v) LA717 after a 7-day culture. Shown is the ratio to the total spheroid number. Spheroids were defined as cell aggregates that had more than five cell nuclei. Data represent means ± SD for 3 independent experiments.

We further investigated how cells are populated in the culture with LA717. As shown in Fig. 1a,b, addition of LA717 to the culture medium successfully dispersed A549 cells and formed uniform spheroids, whereas without LA717 cells formed large clumps that accumulated at the edge of the wells. In addition, it was noted in time-lapse analyses that cells in LA717-containing medium largely stay at the same position during the culture. This is in contrast to the culture without LA717 where the cells move due to the convection of the medium, that causes cell assembly, cell adhesion and formation of clumps (Supplementary Video. S1). The average diameter of A549 spheroids in LA717-containing medium after a 7-day culture was 63 μm, with the mode at 50 μm (Fig. 1c). Scanning electron microscope (SEM) analysis showed a mix of rugged and flat surfaces both covered with microvilli (Supplementary Fig. S1). In agreement with the static culture condition, we confirmed clonal growth in almost all of the spheroids in LA717-containing medium when co-culturing two populations of HEK-293 cells, labelled or not-labelled with a fluorescent marker (Supplementary Fig. S2). These results demonstrate that LA717 causes uniform dispersion of cells and suppresses the medium convection while keeping the medium as liquid, resulting in individual cells forming clonal spheroids. Large-sized spheroids were prevented because of LA717 inhibiting the fusion of spheroids.

Next, we characterised the viscosity, osmotic pressure, absorbance at 450 nm and pH of LA717-containing DMEM by comparing them to those of DMEM controls (with or without water, a vehicle control) and DMEM with 0.4% agarose or 0.03% methyl cellulose as an example of highly viscous medium. The differences in the viscosity between DMEM with and without LA717 were, although statistically significant, not noticeable in the practical handling, as both were able to be handled as a liquid. By comparison, the viscosity of DMEM with agarose was far greater at 0.4%, the concentration employed for soft agar colony formation assays^23^ (Supplementary Table S2). Furthermore, addition of 0.03% methyl cellulose to the medium resulted in a larger increase in viscosity than 0.03% LA717. However, uniform cell dispersion was not induced (Supplementary Tables S1,S2). The data suggest that a slight increase of viscosity by LA717 is unlikely attributed to the cell-dispersing effect. Osmotic pressure was slightly reduced by addition of LA717 or water, likely due to the volume taken by 0.03%. The slight change in pH by additional LA717 was considered well within the range of the buffering capacity DMEM normally has. With regard to the optical transparency, LA717-containing medium showed a similar absorbance to that of DMEM. Hence, addition of LA717 does not compromise practicality in cell culture and medium handling such as cell seeding, reagent addition, microscopic observation and medium changes.

### LA717 improves cell proliferation of cancer cells without serving as scaffold

**Figure 2:**
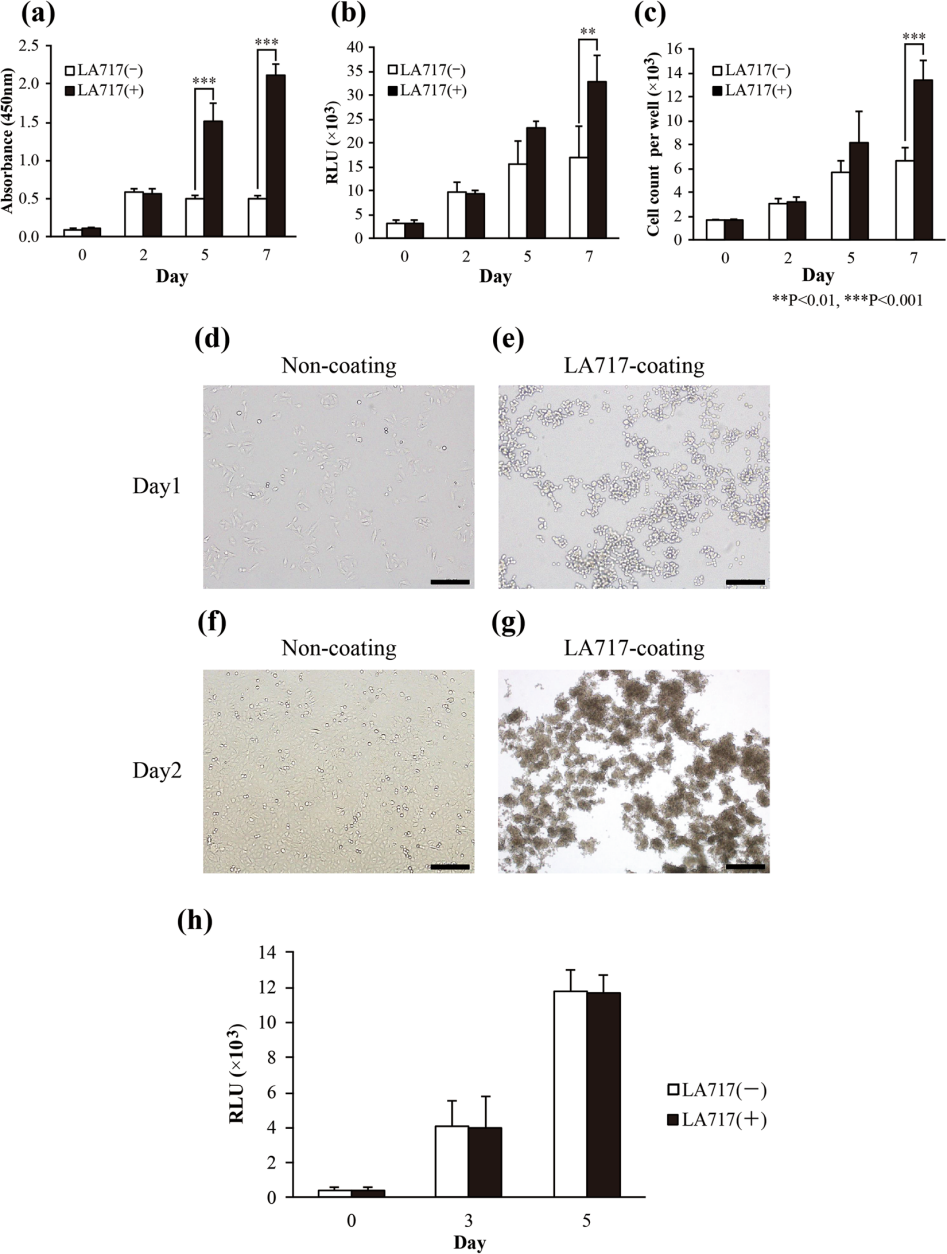
LA717 improves the growth of A549 cells in 3D cultures. **(a–c)** LA717 promotes cell viability. A549 cells were cultured on low-attachment plates without (white columns) or with (black columns) LA717 and analysed with WST-8 (**a**), ATP (**b**), and Trypan blue exclusion assays (**c**). **(d–g)** LA717 does not function as a cell scaffold. A549 cells were grown on cell-attachable plates in the absence or presence of LA717 coating and cultured for 1 (**d**,**e**) and 2 days (**f**,**g**). A549 cells do not adhere to LA717-coated plate and instead form spheroids in 3D. The bars represent 200 μm. **(h)** LA717 has no effect on cell proliferation when cells are grown in 2D on attachable plates. A549 cells were grown in 96-well cell-attachable plates for 1 day, after which time the medium was replaced with DMEM with LA717 and cultured for up to 5 days. The number of live cells was counted using the ATP assay. Y-axis shows Relative Light Unit. In (**a**), (**b**), (**c**) and (**h**), data represent means ± SD for 3 independent experiments. Statistical significance was analysed with Tukey’s test.

When anchorage-dependent cells are liquid-cultured on low-attachment plates, cell proliferation tends to be compromised due to the loss of anchorage and subsequent large aggregate formation. To investigate whether cancer cell dispersion by LA717 impacts cell proliferation, we cultured A549 cells in LA717-containing medium on low-attachment plates for 7 days and examined cell viability, proliferation and cell death using WST-8, ATP and Trypan blue assays. A549 cells grew faster in LA717-containing medium as compared with the absence of LA717, both of which were in the anchorage-independent condition (Fig. 2a–c). The positive effect of LA717 on cell proliferation was also observed in other cancer cell lines T98G, MNNG/HOS, HepG2, A375, HCT116, MDA-MB-231, A431, MCF-7 and SKOV3 (Table 1). By contrast, LA717 had no significant effect on the growth of MIA PaCa-2, HeLa, AGS and LNCap cells (Table 1). Among them, MIA PaCa-2 and AGS did not form spheroids and remained as single cells in the presence or absence of LA717 (data not shown). These results suggest that spheroid forming ability may be one of the factors allowing LA717 to exert its growth-enhancing effect. Other factors that make HeLa and LNCap cells refractory to LA717 are yet to be explored. To further confirm anchorage-independent cell proliferation, we coated plastic plates with LA717 and examined the effect in 2D monolayer cultures. A549 cells did not adhere to the surface of LA717-coated plates, demonstrating that LA717 does not serve as a cell scaffold (Fig. 2d–g). As the medium per se does not contain LA717, the cells aggregated and floated as clumps in the medium (Fig. 2e,g). Furthermore, addition of LA717 to the culture medium on conventional cell-attachable culture plates allowed A549 cells to grow on the plastic surface and didn’t alter the growth rate (Fig. 2h). These results suggest that LA717 does not serve as a scaffold and improves cell proliferation likely by inhibiting the formation of large aggregates which would cause nutrient deficiency.

**Table 1:** Growth of cancer cell lines cultured in the medium with LA717.

Cell name	Tissue	Derivation	Medium	Fold-change	p-value (t-test)
T98G	Brain	Glioblastoma	DMEM/Ham’s F-12	4.1 ± 1.8	<0.01
MNNG/HOS	Bone	Osteosarcoma	EMEM	3.6 ± 1.0	<0.01
HepG2	Liver	Carcinoma	DMEM	3.3 ± 1.0	<0.01
A375	Skin	Melanoma	DMEM	2.5 ± 0.2	<0.01
HCT 116	Colon	Carcinoma	McCoy’s 5 A	2.5 ± 0.6	<0.01
MDA-MB-231	Breast	Adenocarcinoma	DMEM	2.3 ± 0.3	<0.01
A431*	Skin	Epidermoid carcinoma	EMEM	2.4 ± 0.5	<0.01
MCF-7	Breast	Adenocarcinoma	EMEM	2.2 ± 0.3	<0.05
A549	Lung	Carcinoma	DMEM	2.1 ± 0.1	<0.01
SKOV3*	Ovary	Adenocarcinoma	McCoy’s 5 A	2.1 ± 0.3	<0.01
MIA PaCa-2	Pancreas	Carcinoma	DMEM	1.3 ± 0.2	0.11
HeLa	Cervix	Adenocarcinoma	DMEM	1.3 ± 0.1	0.07
AGS	Stomach	Adenocarcinoma	Ham’s F-12	1.1 ± 0.0	0.26
LNCap	Prostate	Adenocarcinoma	RPMI-1640	0.7 ± 0.2	0.51

The listed cell lines were cultured in 3 wells of 96-well low-attachment plates in the indicated medium without (Blank) or with 0.030% (w/v) LA717 for up to 7 days. The numbers of cultured cells were counted using an ATP assay method on day 7 and the fold-change (Cell number in LA717-containing medium / Cell number in Blank) was calculated. Data represent means ± SD for 3 independent experiments.

*100 ng/mL of HB-EGF (Heparin-binding EGF-like growth factor) was added for cell growth.

### LA717 has little effect on gene expression profile in the 3D culture

**Figure 3:**
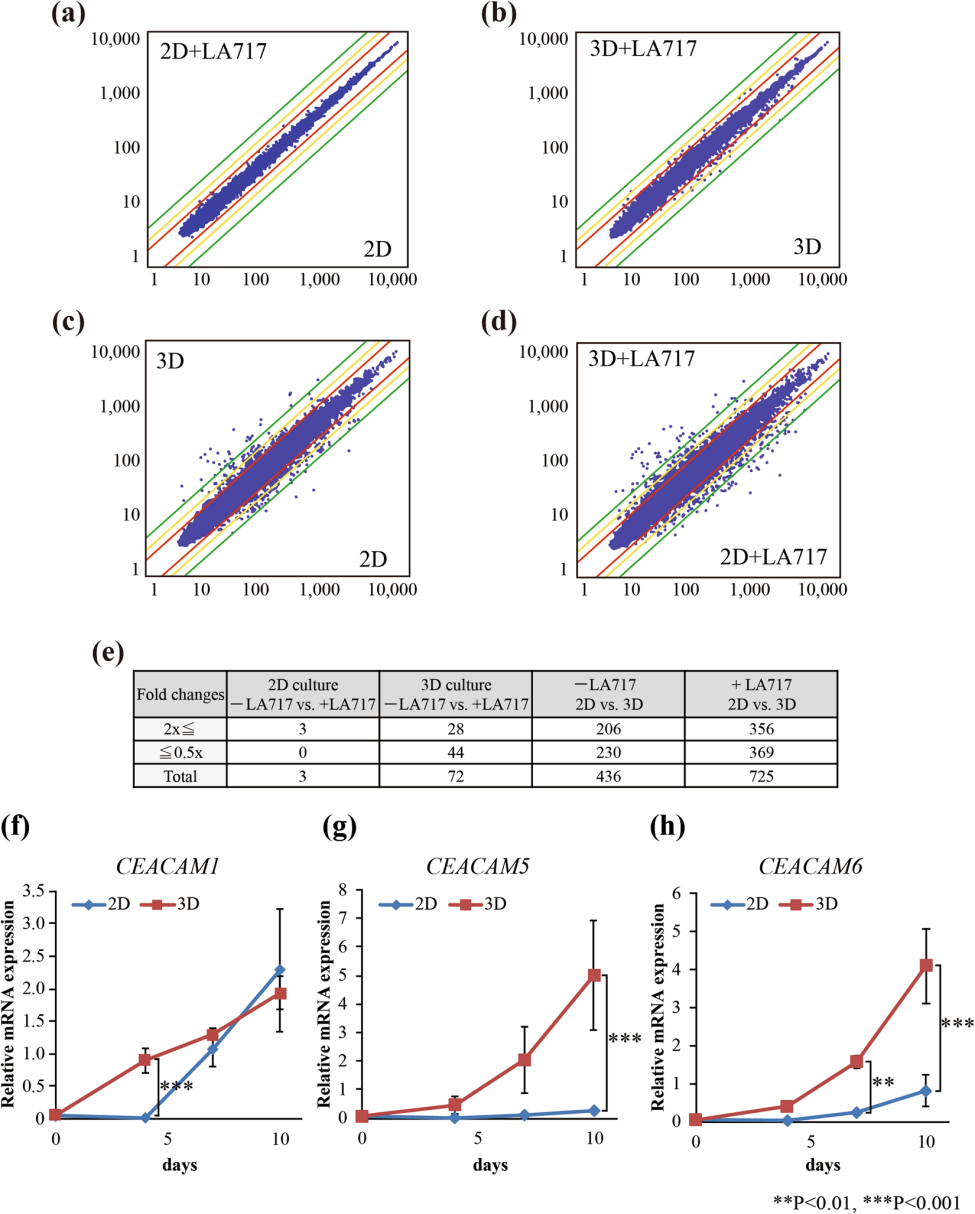
Microarray analyses on gene expression changes caused by 2D vs 3D and by LA717. **(a-d)** A549 cells were cultured with or without 0.030% (w/v) LA717 for 7 days under 2D or 3D conditions. Comparisons were made in indicated combinations. Scatter plots represent individual genes’ expression levels in each condition. Red, yellow and green lines indicate the range of ×2, ×3, ×5 differences. **(e)** The number of genes with greater than twofold changes in each comparison. **(f**–**h)** Real-time PCR analyses of *CEACAM1* (**f**), *5* (**g**) and *6* (**h**) gene expression in 2D and 3D cultures. A549 cells were cultured on 96-well cell-attachable (2D, blue) or low-attachment plates with 0.030% (w/v) LA717 (3D, red) for 4, 7 and 10 days. Data represent mean expression ± SD normalised by *GAPDH* gene for 3 independent experiments. Statistical significance was analysed with Tukey’s test.

We next performed DNA microarray analyses to evaluate the effect of LA717 on the global transcription. Figure 3a–d shows scatter plots on which each dot represents the expression level of individual genes in A549 cells after culturing for 7 days in four different conditions; 2D culture on cell-attachable plates with or without LA717, and 3D culture on low-attachment plates with or without LA717. Comparisons were made in four combinations as shown in Fig. 3a–d and the number of genes with two-times greater changes in given comparison were shown in Fig. 3e. Consistent with the result of Fig. 2d–g, comparison between 2D cultures with and without LA717 showed little difference (3 genes altered by more than twofold among the 39130 genes examined, Fig. 3a,e). Comparisons between 2D and 3D detected many gene expressions altered, in both absence and presence of LA717 (436 and 725 genes out of 39130 genes, respectively) (Fig. 3c–e). In contrast, comparison between the presence and absence of LA717 in 3D cultures resulted in 72 out of 39130 genes altered (Fig. 3b,e). This was far lower compared to the 2D vs 3D differences noted in Fig. 3c–e, thus, the impact LA717 gives on gene expression profile is relatively low.

Supplementary Table S3 shows the twenty most up- or down-regulated protein-coding genes in cells cultured in 3D in the presence of LA717 compared to the 2D culture. Of those, changes in the expression of carcinoembryonic antigen-related cell adhesion molecule (*CEACAM*) *5* and *6* were confirmed using real-time quantitative PCR (Fig. 3g,h), along with *CEACAM1* as a control (Fig. 3f). *CEACAM5* and *6* were up-regulated over time in the 3D condition with LA717. In contrast, there was no significant difference in *CEACAM1* expression between cells cultured in 2D and 3D after 7–10 days, although on day 4 the expression was higher in 3D (Fig. 3f). The data is consistent with the report that regulation of *CEACAM1* gene expression was different from that of *CEACAM5* and *6* genes^24^. Taken together, while 2D versus 3D conditions give a strong impact on gene expression profile, additional LA717 has relatively little effect on gene regulation.

### Anti-cancer drug evaluation in 3D culture with LA717

**Figure 4:**
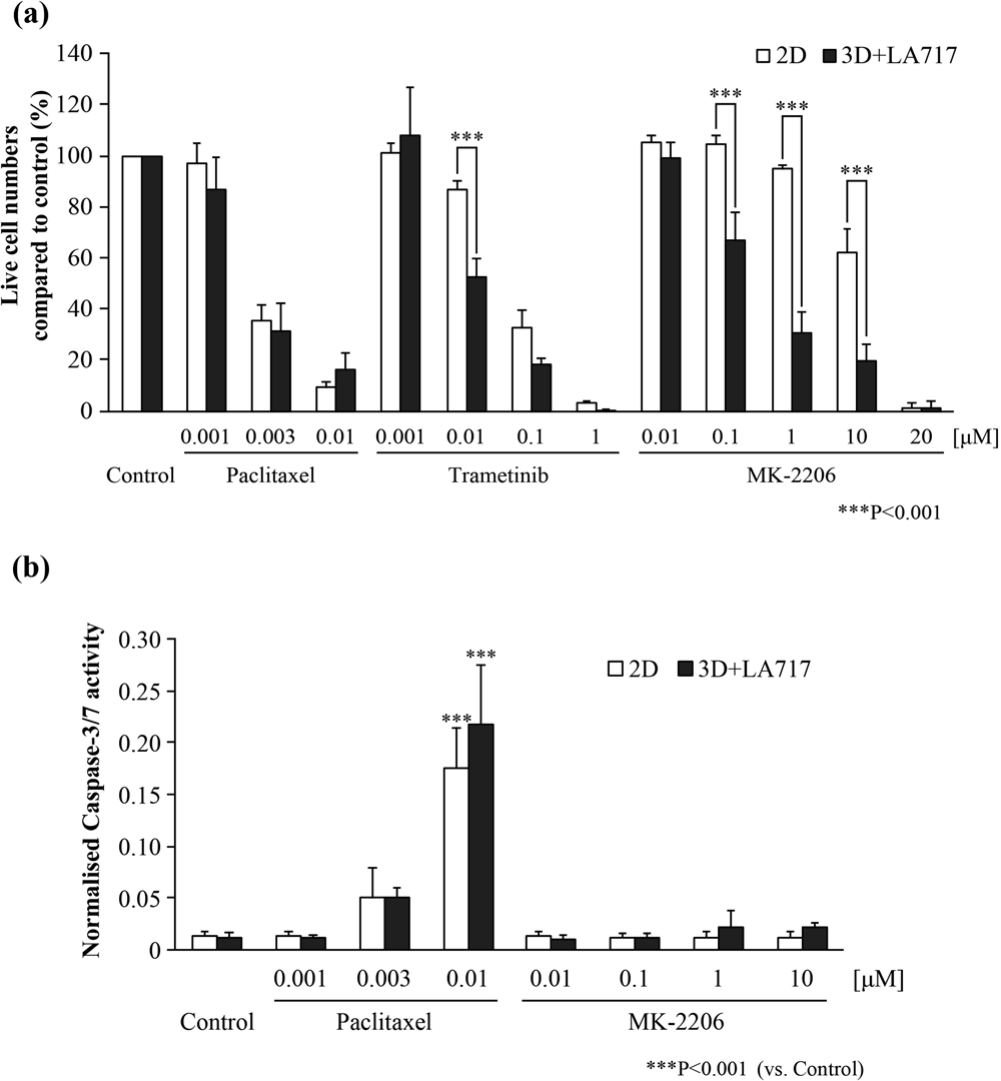
Application of 3D culture with LA717 to anti-cancer drug evaluation. **(a)** A549 cells were grown at 1000 cells/well in 96-well cell-attachable plates in DMEM (2D) or in low-attachment plates with 0.030% (w/v) LA717 (3D). After 1 day of culture, anti-cancer drugs or DMSO (Control) were added. The cells were further cultured for 7 days and the cell number was counted using ATP assay. Data represent means ± SD of 3 independent experiments. Statistical significance was analysed with Tukey’s test. **(b)** Anti-cancer drug-treated cells were prepared as in (**a**). The Y-axis indicates the Caspase 3/7-Glo value (apoptosis activity) normalised to the ATP value (cell number). Statistical significance was analysed with Dunnett’s test (vs. Control). Data represent means ± SD of 3 independent experiments.

To assess the benefit of LA717 for drug screening, we examined the sensitivity of A549 cells to anti-cancer drugs in the presence or absence of LA717 on low-attachment plates. We chose the anti-mitotic drug Paclitaxel^25^ and the MEK1/2 inhibitor Trametinib^26^ as established anti-cancer drugs, and an AKT inhibitor MK-2206^27^ as a candidate anti-cancer drug, for the cytotoxicity analyses. Figure 4a and Supplementary Table S4 show the sensitivity of A549 cells to Paclitaxel, Trametinib and MK-2206 in 2D on cell-attachable plates and in 3D on low-attachment plates in the presence of LA717. As for Paclitaxel, the effect of inhibiting cell survival was similar in both 2D and 3D cultures. In contrast, Trametinib and MK-2206 were shown to be more effective in 3D than in 2D. In fact, the value of IC50 of MK-2206 on inhibition of cell growth was more than 10 times different between 2D and 3D cultures (Table S4). The result demonstrates that the effect of both Trametinib and MK-2206 (the drugs proven to be effective *in vivo*^26,27^), is masked in the 2D assessment, but is clearly manifested in 3D cultures with LA717. The data also suggest that the action of certain signal transduction inhibitors can be elucidated at lower concentrations in 3D culture.

We also examined caspase activity on Paclitaxel and MK-2206 to test the specificity of drug action in the culture. As expected as an anti-mitogenic inhibitor^25^, Paclitaxel induced caspase activity in a dose-dependent manner in both 2D and 3D with LA717 (Fig. 4b). In contrast, MK-2206, which inhibits cell proliferation independent of caspase^28,29^, did not induce caspase activity in 2D or 3D. The data demonstrates that the 3D culture with LA717 faithfully presents the drug function and hence is likely a useful method for evaluating the activity of anti-cancer drugs.

Next, we examined the impact of serum on the efficacy of Paclitaxel and MK-2206 using 2D and 3D cultures, as serum-free media are often used to evaluate anti-cancer drugs on cancer stem cells. We employed the MCF-7 human breast adenocarcinoma cell line for this experiment because MCF-7 cells exhibit more vigorous growth in the absence of serum in the 3D condition than A549 cells (data not shown). Supplementary Figure S3 and Table S5 show the sensitivity of MCF-7 cells to Paclitaxel and MK-2206 when cultured in 2D or 3D with or without LA717. Similarly to what is seen in A549 in Fig. 4a, the cytotoxic effect of MK-2206 on MCF-7 was more prominent in 3D than in 2D in the presence of serum, although MCF-7 showed a slightly higher sensitivity to MK-2206 in 2D (Fig. S3) compared to A549. The effect of Paclitaxel was similar in both 2D and 3D on MCF-7, as was seen on A549 cells. In the serum-free condition, however, the effect of 3D culture with LA717 in exhibiting the cytotoxicity of MK-2206 was no longer observed, as both 2D culture and 3D culture with LA717 showed a similar efficacy of MK-2206 (Fig. S3). Hence the effect of 3D culture on the toxicity of MK-2206 on MCF-7 is serum-dependent.

Next, we compared the effect of MK-2206 between 3D culture with LA717 and conventional soft agar assay (Fig. S4). The efficacy of MK-2206 on A549 cell growth in 3D culture with LA717 was similar to that in the soft-agar culture. In contrast, 3D cultures without LA717 or 2D cultures showed lower efficacy compared to that with LA717. The result indicates that the 3D culture with LA717 is comparable to soft agar cultures with the added benefit of practicality in handling and microscopic observation. The lower sensitivity of cells cultured under 3D conditions without LA717 might be due to decreased drug permeability of large sized-spheroids.

### 3D culture with LA717 increases feasibility in high-content analysis/screening system

**Figure 5:**
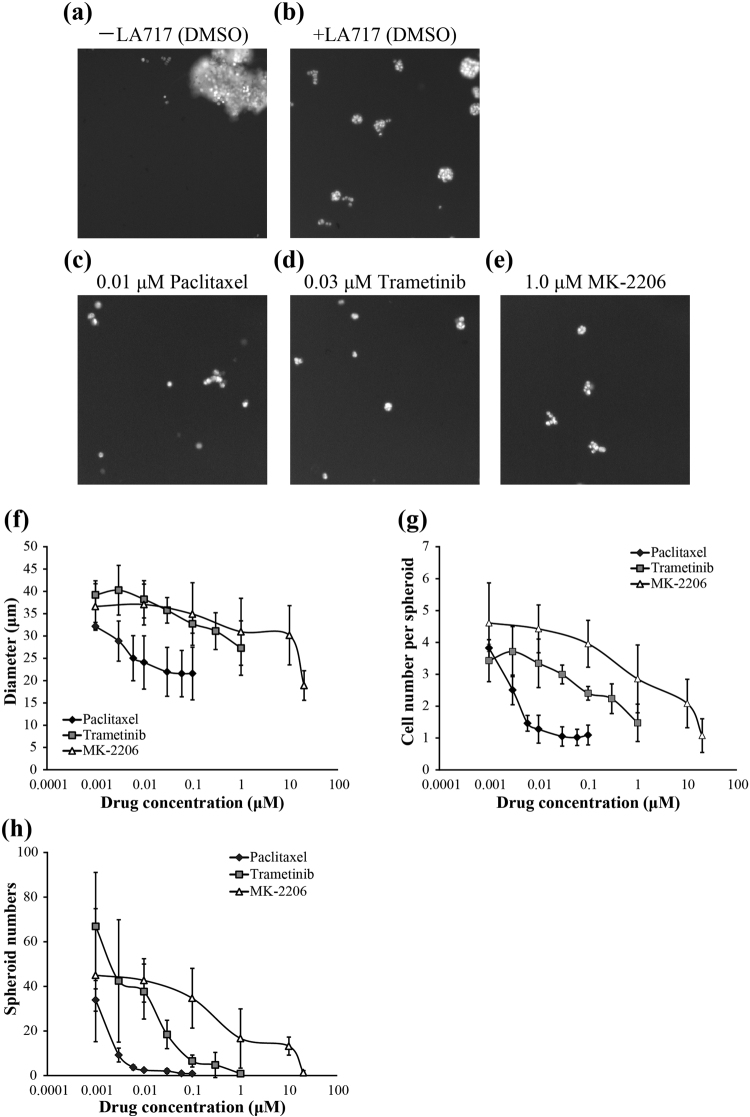
The high content analysis of A549 cells treated with anti-cancer drugs. Anti-cancer drug-treated cells were prepared as in Fig. 3(a). (**a–e**) Fluorescent images of A549 spheroids stained with Hoechst 33342. A549 spheroids were cultured without (**a**) or with 0.030% (w/v) LA717 (**b**–**d**). They were treated with DMSO (**a, b**; control), 0.01 μM Paclitaxel (**c**), 0.03 μM Trametinib (**d**) or 1.0 μM MK-2206 (**e**) for 7 days. **(f–h)** Dose-dependent effect of anti-cancer drugs on A549 cell growth. Graphs show the diameter of spheroids (**f**), the cell number in an A549 spheroid (**g**), and the total number of spheroids (diameter ≥50 μm) (**h**). Data represent means ± SD for 3 independent experiments.

**Figure 6:**
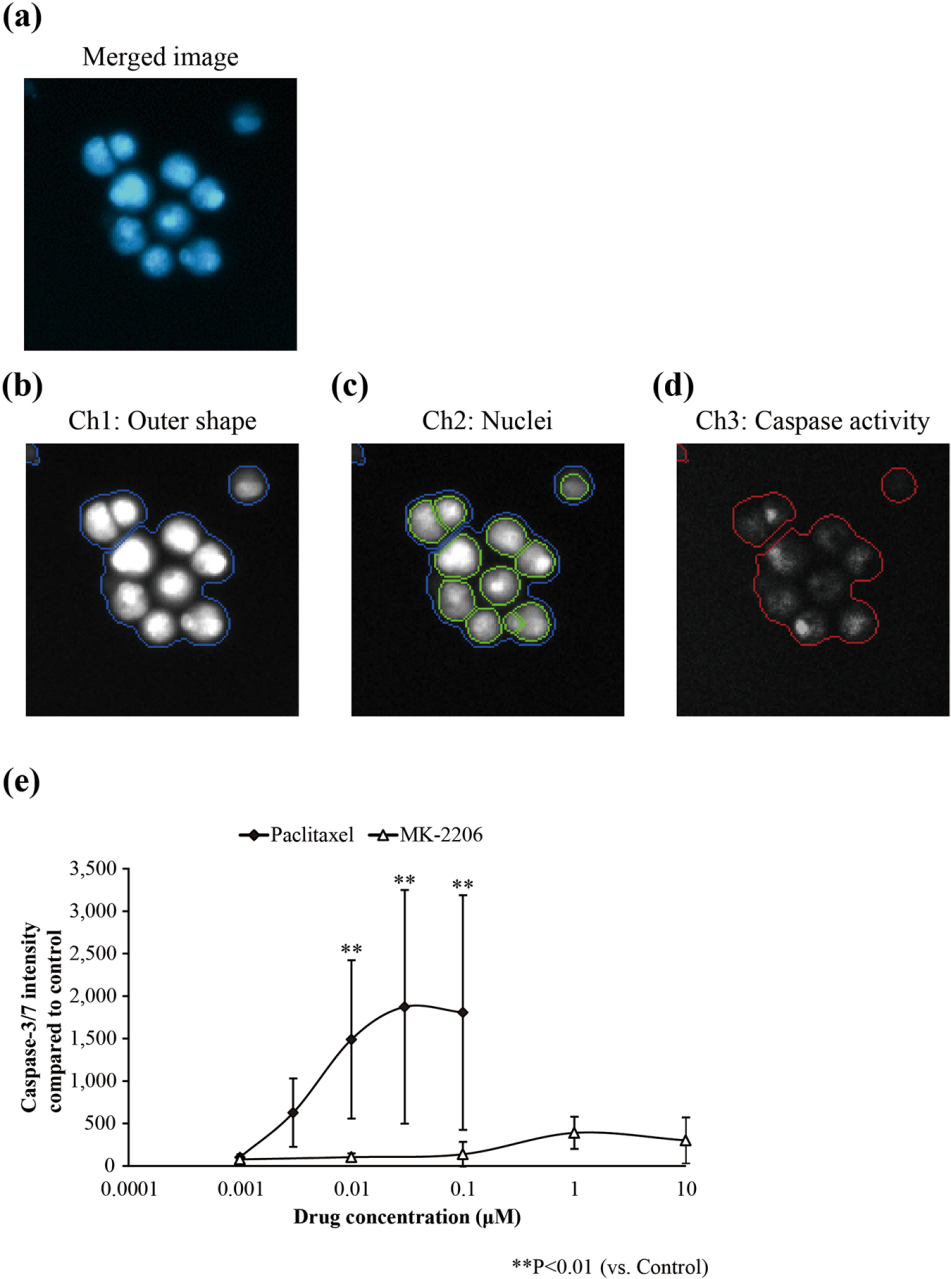
Caspase-3/7 assay on anti-cancer drugs-treated A549 cells using HCA. **(a**–**d)** Fluorescent images of A549 spheroid stained with Hoechst 33342 and CellEvent^TM^ Caspase-3/7 Green Detection Reagent, taken using the Cellomics® ArrayScan® VTI. Images are the merged image (**a**), the outline of cells (b, Ch 1), nuclei (c, Ch2) and caspase-3/7 activity (d, Ch3). **(e)** Detection of activated Caspase-3/7 in A549 spheroids (diameter ≥50 μm). The intensity of CellEvent^TM^ staining was measured within the area outlined in (**b**) (blue line in (**b**) and red line in (**d**)). Y axis shows the intensity of CellEvent^TM^ Caspase 3/7 Green Detection Reagent compared to the control (DMSO-treated spheroids). Statistical significance was analysed by Dunnett’s test (vs. Control). Data represent means ± SD of 3 independent experiments.

We next investigated whether LA717 is suitable for drug evaluation with automated cell imaging apparatus, that is, high-content analysis/screening (HCA/HCS). As shown in Fig. 5a, A549 cells grown in low-attachment 96-well plates in the absence of LA717 formed large clumps that tend to stay at the periphery of the well, hence the automated imaging system often fails to capture such cell images. LA717-containing medium provided homogeneous dispersion of A549 cell spheroids (Fig. 5b; see also Fig. 1b,c). The growth inhibitory effect of anti-cancer drugs was reflected on the size and number of the spheroids as well as the cell number in each spheroid, that were all readily detectable by the imaging analyser thanks to the clear medium and the cells staying at the single focal plane (Fig. 5c–e). As shown in the quantitative analysis in Fig. 5f–h, Paclitaxel, Trametinib and MK-2206 decreased all parameters; spheroid diameter, the cell number in individual spheroids and the number of spheroids, in a concentration dependent manner. The IC50 values obtained by the imaging analyser were comparable to those obtained by the ATP assay (Supplementary Table S4). In addition, the automated image analyser was able to detect apoptosis inside the spheroid of Paclitaxel-treated A549 cells (Fig. 6a–d) by Paclitaxel, while no significant caspase activity was detected in cells treated with MK-2206 (Fig. 6e). This was in consistent with the observation shown in Fig. 4b. These data indicate that the 3D culture with LA717 is suitable for the HCA/HCS.

To further evaluate the use of LA717 in a large-scale screening, we performed a HTS/HCS using 384-well low-attachment plates and LA717-containing medium. The coefficient of variation (CV) and the Z’-factor for the plate, when counting A549 cell number using ATP assay after 5 days of culture, were 10.76 ± 1.94 and 0.67 ± 0.05, respectively. Given that the Z’-factor is above 0.5, the statistical dispersion of cell growth per well is in an acceptable range. Supplementary Figures S4 and S5 show the growth-inhibitory effect of anti-cancer drugs, Paclitaxel, Trametinib and MK-2206, that were assessed using the 384-well low-attachment plates with LA717-containing medium with ATP assay (Supplementary Figure S4) and imaging analyser (Supplementary Figure S5). These results demonstrate that LA717-containing 3D culture is suitable for HTS/HCS with 384-well plates, which enables us to perform large-scale screening.

## Discussion

We have demonstrated that the LA717 offers a new type of 3D culture method that has unique features and advantages. The main feature is that, when cells are seeded in the LA717-containing medium following dissociation, the cells uniformly disperse in the medium and does not gather to form clumps. This is due to the feature of LA717 that (1) it prevents new cell adhesion while allowing to maintain existing cell adhesion after cell division and (2) it keeps the movement of the liquid to a minimum by occupying the space, hence convection is kept minimum and the cells and spheroids stay at the same position. These features not only prevent cells from forming clumps and become necrotic, but also make the analyses effective, as the spheroids are relatively homogeneous in size over time and readily exposed to the medium. The system is especially useful for studying the effect of exogenous factors in the medium, as we have shown higher sensitivities to some of anti-cancer drugs compared to the conventional 2D system. The fact that the cells and spheroids tend to stay still in their position is advantageous for capturing time-lapse movies. The high solubility of LA717 to the medium and its transparency are other advantages for the imaging analysis. In addition, the low viscosity of the medium enables practical handling of the medium. Thus, LA717 makes a virtual 3D culture possible in a liquid form.

Another unique feature is that LA717 does not serve as a scaffold. It is therefore more suitable for cancer cell studies such as drug screening and the evaluation, and not for non-cancerous cells such as epithelial cells and fibroblasts that require scaffolds. For these cell types, adding a certain amount of gel matrix together with LA717 will allow formation of epithelial cysts and spindle shaped-morphology, respectively, keeping some space for additional exogenous factors to test. Thus, LA717 has further potential applications yet to develop. At present, the feature of LA717 may be best presented in anti-cancer drug tests as shown in this study, where cells do not require scaffold and form homogeneous spheroids in the liquid medium, allowing the cells to be consistently exposed to dissolved drugs. Large-scale screening of anti-cancer drugs with HCA/HCS is also a well-suited application of LA717, in which transparent and non-viscous medium makes auto-imaging and liquid-handling possible. It is anticipated that LA717 promotes applications of HCS to the increasingly demanding field such as personalised medicine using patients’ tumor cells. Cells’ sedimentation and even distribution on the plate is a definite advantage not only for HCA/HCS but also for conventional time-lapse imaging.

One of the striking results in this study is that the cytotoxic effect of the AKT inhibitor MK-2206 is only evident in the 3D culture and not in 2D, whereas that of Paclitaxel, an anti-mitogenic drug, is similar in 2D and 3D. Our result is consistent with the report by Riedl *et al*. that 3D-cultured spheroids show augmented response to AKT-mTOR or MEK inhibitors compared to 2D-cultured cells^30^. In addition, we found that elimination of serum from the medium weakens the growth-inhibitory effect of MK-2206 in the 3D condition while the effect of Paclitaxel was not affected. It would be interesting to know whether drugs working on signal transduction pathways tend to show more prominent effects in 3D cultured cells than in 2D, and whether the similar effects between 2D and 3D is a common feature for anti-mitogenic drugs. It is noteworthy that Trametinib and MK-2206 have been reported to be of relatively low efficacy on cell growth inhibition compared to anti-mitogenic drugs when using 2D culture methods^31–33^. It is possible that the *in vivo* effect of drugs that target AKT or MEK is masked in conventional 2D cultures. In addition, it has been reported that 2D cell cultures induce phosphorylation of ERK1/2 and AKT in cancer cells compared to tumours xenografted in mice, thus resulting in high activity within the pathways and low sensitivity to the blockers^30,34^. In this respect, anti-cancer assay models employing 3D cultures with LA717 are more relevant to *in vivo* conditions compared to those employing 2D cultures.

The analysis of the gene expression profile revealed that there was a relatively small difference between the presence and absence of LA717 in 3D, despite the apparent difference in the shape of the aggregate. This might be because the main difference caused by the presence or absence of LA717 is likely cell necrosis, which does not involve transcriptional regulation, as is evident in apoptosis. In this regard, the fact that LA717 does not act as a microenvironmental factor is another benefit, allowing researchers to add exogenous factors of their choice for examination.

Agar has conventionally been used for soft agar colony formation assays which evaluate the ability of cells to grow in anchorage-independent conditions^35^. The principle of LA717 is similar to this, utilising the nature of agar not serving as a scaffold. Indeed, there was little difference in the efficacy of MK2206 on cancer cell growth between 3D cultures with LA717 and in soft-agar. The major difference is that LA717 is highly soluble, achieved by the low molecular weight and optimal concentration of it. Therefore, LA717 can be used similarly to normal agar for cell culture with the advantage of being able to handle it as a liquid. In this study, application of 3D cultures with LA717 was limited to transformed cells that have the capacity to perform anchorage-independent growth as one of the hallmarks of cancer cells. However, using 3D culture with LA717 may also be useful to examination of non-transformed cells, such as ES/iPS cells, that grow anchorage-independently^36^.

In conclusion, a novel 3D culture method using LA717 improves the growth of spheroid-forming cells, promising it as an effective and sensitive assay approach for detecting changes in cell proliferation, function and drug sensitivity. LA717 enables 3D culture to be a practical, robust and reproducible method, which promotes the effective performance of large-scale assays as well as cell biological studies.

## Materials and Methods

### Compounds and reagents

Low molecular weight (Ultra-agar Ina, average MW 43,000) and standard (S-6, average MW 290,000) agar was purchased from Ina Food Industry Co., Ltd. (Nagano, Japan). To prepare agar stock solution for cell cultures, agar was suspended in pure water to 1.0% (w/v) and dissolved by stirring at 90 °C. The aqueous solution was sterilized at 121 °C for 20 minutes by autoclave. The solution was then added to cell culture media at given concentrations with stirring at room temperature.

Trametinib and MK-2206 were obtained from Santa Cruz (Texas, USA). Paclitaxel was purchased from Wako Pure Chemical Industries (Osaka, Japan).

### Cancer cell lines

All human cancer cell lines used in this study were commercially available. The human cancer cell lines A549, MDA-MB-231, SKOV3, HCT116, MCF-7, A431, T98G, AGS, HeLa and HepG2 were obtained from DS Pharma Biomedical (Osaka, Japan). Other cell lines A375, MNNG/HOS, MIAPaCa-2 and LNCap cells were obtained from American Type Culture Collection (Virginia, USA). A549, A375, HeLa, HepG2, MDA-MB-231 and MIA PaCa-2 cells were cultured in Dulbecco’s modified Eagle’s medium (DMEM) with 10% fetal bovine serum (FBS). HCT116 and SKOV3 cells were cultured in McCoy’s 5 A medium with 10% FBS. MCF-7, MNNG/HOS and A431 cells were cultured in Eagle’s Minimum Essential Medium (EMEM) with 10% FBS. LNCap cells were cultured in Roswell Park Memorial Institute Medium 1640 (RPMI1640) with 10% FBS. These media were supplemented with a 1% penicillin-streptomycin mix (Sigma-Aldrich, Missouri, USA). Cells were all cultured at 37 °C in a humidified atmosphere with 5% CO_2_.

### Cell culture using medium with LA717

Each human cancer cell line was seeded at a density of 5,000-20,000 cells/mL into the appropriate medium containing 0.03% (w/v) of LA717, and was dispensed into 96-well flat-bottom low-attachment plates using 100–200 μL/well. For the monolayer culture method (2D), each cancer cell line was inoculated at a density of 2,000–15,000 cells/mL in the medium without LA717, and dispensed into 96-well flat-bottom cell-attachable plates (Corning Incorporated) using 100–200 μL/well. These plates were cultured unstirred for 2–10 days.

For the anti-cancer drug test, medium containing a 10-fold concentration of various anti-cancer drugs and 0.03% (w/v) LA717-containing cell culture media were combined at the ratio of 1:9.

### Cell proliferation assay

For the cell proliferation assay, cells were counted as follows: for the WST-8 assay, WST-8 solution (Dojindo Laboratories, Kumamoto, Japan) was added to the cell culture at the 1/10 of the volume and incubated at 37 °C for 100 minutes. Viable cells were counted by measurement of the absorbance at 450 nm using the absorbance spectrometer SpectraMax® 190 (Molecular Devices, California, USA). For the ATP assay, an equal volume of ATP reagent (CellTiter-Glo® Luminescent Cell Viability Assay; Promega, Wisconsin, USA) was added to the culture medium and the luminescence intensity (Relative Light Unit (RLU) value) was measured using FlexStation3 (Molecular Devices, California, USA). For the trypan blue exclusion assay, spheroids were dissociated to single cells by trypsin-EDTA treatment for 10 min and then cells were stained with an equal volume of 0.4% (w/v) trypan blue solution (Life Technologies, California, USA). Viable cells were counted using an automated cell counter (Bio-Rad, California, USA, TC20).

### Cell culture on LA717-coated plates

LA717 aqueous solution (1.0% (w/v), 500 μL/well) was added to 12-well flat bottom plates (Corning Incorporated, #3513). After incubation for 60 minutes at room temperature, A549 cells were plated on the LA717-coated plates at a density of 100,000 cells/2 mL/well and were cultured for 5 days. The cultured cells were observed under the light microscope at 100-fold magnification (Olympus, Tokyo, Japan).

### Microarray analysis

Total RNA was isolated from 7-day cultured A549 cells using RNeasy Kit (Qiagen, California, USA) according to manufacturer’s protocol. Each RNA sample was analysed using a GeneChip® Human Gene 2.0 ST Array (Affimetrix, California, USA and Kurabo, Osaka, Japan). Up- and down-regulated genes with at least two fold changes were selected using DNA MicroArray Viewer Software (Kurabo). All data are MIAME compliant and the raw data were deposited in the Gene Expression Omnibus (accession number; GSE103702, GSE103845).

### Real-time PCR

Real-time PCR was carried out for 40 to 45 cycles of 15 seconds at 95 °C and 1 minutes at 60 °C using ABI PRISM® 7700 Sequence Detector (Applied Biosystems, California, USA). All Taqman primers and probes were obtained from Applied Biosystems; *GAPDH* (Hs02758991_g1), *CEACAM1* (Hs00989786_m1), *CEACAM*5 (Hs00944025_m1) and *CEACAM*6 (Hs03645554_m1).

### Apoptosis assay

Caspase 3/7 reagent (Caspase-Glo® 3/7, Promega) was added to the culture medium after 4 days of cell culture as appropriate. Following incubation for 1 hour at room temperature, luminescence intensity (RLU value) was measured using FlexStation3®. The values were normalised to the ATP value.

### High content analysis

For quantitative analyses for the size and distribution of spheroids and anti-cancer drug evaluation, spheroids were stained with 10 μg/mL Hoechst 33342 in phenol red-free DMEM. For caspase-3/7 assay, the staining solution which consisted of DMEM without phenol red, 2 μM CellEvent™ Caspase-3/7 (ThermoFisherScientific, Massachusetts, USA) and 10 μg/mL Hoechst 33342 was added to the culture medium.

After incubation for 45–60 min at 37 °C, fluorescent images were automatically taken, stored, and analysed by Cellomics®ArrayScan® VTI. Ten fields were analysed in each well with a × 10 objective. The morphology algorithm in ArrayScan® VTI was used to detect the saturated fluorescence of Hoechst 33342 that allowed us to identify the outline of spheroids at channel 1, whilst the fluorescence of Hoechst 33342 identifying cell nuclei was detected at channel 2. The fluorescence of CellEvent™ Caspase-3/7 as activated caspase-3/7 was detected at channel 3 within the area gated by the outline of spheroids.

### Statistical analysis

All results are presented as the mean ± standard deviation (SD). Statistical significance was analysed with Student’s t-test, Dunnett’s test, or Tukey’s test as indicated in each legend, using EXSAS ver. 7.1.6.1 (Arm Systex, Osaka, Japan). The level of significance was set at 0.05 unless stated otherwise.

### Data availability

The datasets generated during and/or analysed during the current study are available from the corresponding author on reasonable request.

## Electronic supplementary material

Supplementary Information

Supplementary Video
