# Supplementary material for: Novel 3D Liquid Cell Culture Method for Anchorage-independent Cell Growth, Cell Imaging and Automated Drug Screening
Source: Sci Rep. 2018 Feb 26;8:3627. doi: 10.1038/s41598-018-21950-5 (PMC5827526; doi:10.1038/s41598-018-21950-5)
Supplement: Supplementary file 1 — Supplementary Information [file 41598_2018_21950_MOESM1_ESM.pdf]

Supplementary Information

Novel 3D Liquid Cell Culture Method for Anchorage-independent Cell Growth, Cell Imaging and Automated Drug Screening

Natsuki Abe-Fukasawa[1], Keiichiro Otsuka[1], Ayako Aihara[1], Nobue Itasaki[2], and Taito Nishino[1*]

[1]Biological Research Laboratories, Nissan Chemical Industries LTD., Saitama, Japan
[2]Faculty of Health Sciences, University of Bristol, Bristol, United Kingdom

*Corresponding author: Taito Nishino, Ph.D.

Biological Research Laboratories, Nissan Chemical Industries LTD., 1470, Shiraoka, Shiraoka-shi, Saitama, 349-0218, Japan

TEL: +81-480-92-2513, FAX: +81-480-92-2516, E-mail: nishino@nissanchem.co.jp

Supplementary Table S1. Screening of polysaccharides examined for 3D cell cultures in this study

Polymers	Concentration %(w/v)	Aggregates of polysaccharides	States of A549 cell spheroids	
			Uniform cell dispersion	Suspension vs. Sedimentation
Low molecular weight agar	<0.03	No	No	Sedimentation
	0.03	No	Yes	Sedimentation
	0.05<	No	Yes	Suspending
Standard molecular weight agar	<0.03	Yes	No	Sedimentation
	0.03	Yes	Yes	Sedimentation
	0.05<	Yes	Yes	Suspending
Agarose	<0.03	Yes	No	Sedimentation
	0.03	Yes	Yes	Sedimentation
	0.07<	Yes	Yes	Suspending
Low-melting agarose	<0.1	Yes	No	Sedimentation
	0.1	Yes	Yes	Sedimentation
	0.1<	Yes	Yes	Suspending
κ-Carrageenan	≦0.05	No	No	Sedimentation
Xanthan gum	≦0.1	No	No	Sedimentation
Gellan gum	<0.01	No	No	Sedimentation
	0.01≦	No	Yes	Suspending
Methyl cellulose	<0.6	No	No	Sedimentation

Low-molecular weight agar (Ina) and Standard-molecular weight agar (S-6) were purchased from Ina Food Industry (Nagano, Japan). Agarose (Agarose S) was obtained from Nippon-Gene (Japan). Low-melting agarose and Methyl cellulose (cP1500) were obtained from Sigma-Aldrich (Missouri, USA). κ-carrageenan, xanthan gum and Gellan gum (Kelcogel CG-LA) were purchased from Sansho (Osaka, Japan). Each polysaccharide was suspended in pure water to a concentration of 0.1-1% (w/v) and

dissolved by stirring at 80-90 °C with the exception of methyl cellulose which was dissolved by stirring on ice. The aqueous solutions were further diluted in DMEM to the final concentration. After preparation, the medium in a tube was observed to detect the presence of aggregate of polysaccharide.

A549 cells were seeded at a density of 20,000 cells/mL in the above polysaccharides containing medium and dispensed into 96-well flat-bottom low attachment plates using 100 μL/well. The state of A549 spheroid was observed after 7 days of culture; dispensed homogeneously or not, and sedimented or suspended in the medium.

Supplementary Table S2. The physical characteristic of LA717 containing medium

Sample	Viscosity (mPa・S)	Osmotic pressure (mOsm/kg)	Absorbance O.D.450nm	pH
Control: DMEM	0.729±0.001	384.2±2.049	0.327±0.006	7.342±0.013
0.03% LA717 containing DMEM	0.785±0.014*	371.8±2.490*	0.323±0.004	7.370±0.012*
0.03% sterile water (vehicle) containing DMEM	0.729±0.001	370.2±0.447*	0.322±0.003	7.400±0.017*
0.4% Agarose containing DMEM	1.879±0.018*	Not tested	Not tested	Not tested
0.03% Methyl cellulose containing DMEM	0.850±0.019*	Not tested	Not tested	Not tested

*P<0.05 (vs. Control)

The viscosity of the medium was measured using the rolling-ball viscometer, Lovis 2000 M (Anton Paar, Graz, Austria) at 37 °C. A glass capillary tube (1.59 mm) containing a steel ball was used with an angle of 70°. The osmolarity, absorbance and pH of the medium was measured by using the Osmometer (VOGEL, Giengen, Germany), FlexStation3® (Molecular Devices, California, USA) and pH METER F-72 (HORIBA, Kyoto, Japan).

DMEM containing 0.4% agarose or 0.03% methyl cellulose, frequently used for anchorage independent cell growth tests such as colony assays, was employed as an example of highly viscous medium. Agarose (Lonza, Basel, Switzerland; SeaPlaque™ Agarose) was dissolved in water at 1.2% (w/v) by heating with a microwave oven.

Aqueous solution of Methyl cellulose (Sigma-Aldrich, Missouri, USA) was prepared as described in Table S1. Equal volumes of the agarose or methyl cellulose solution, 2X DMEM prepared from powdered medium (Thermo Fisher Scientific, Massachusetts, USA) and DMEM (Wako Pure Chemical Industries, Osaka, Japan) were mixed. Statistical significance was analysed by Tukey's test. Data represent means ± SD of 5 independent samples.

Supplementary Table S3. Twenty most up- or down-regulated genes in LA717-treated A549 cells

Up-regulated gene	Description	Fold change (vs. 2D)
LOC93432	maltase-glucoamylase (alpha-glucosidase)	25.69±8.12
CEACAM5	carcinoembryonic antigen-related cell adhesion molecule 5, transcript variant 2	21.51±7.11
PLEKHS1	pleckstrin homology domain containing, family S member 1, transcript variant 2	13.96±4.23
KRT20	keratin 20	13.56±1.32
CDH17	cadherin 17, LI cadherin (liver-intestine), transcript variant 2	10.00±1.32
CD22	CD22 molecule, transcript variant 2	9.28±1.26
VTCN1	V-set domain containing T cell activation inhibitor 1, transcript variant 2	8.49±0.41
AGR3	anterior gradient 3	8.41±1.68
BPIFB1	BPI fold containing family B, member 1	7.76±1.15
CYP4F11	cytochrome P450, family 4, subfamily F, polypeptide 11, transcript variant 2	7.30±0.89
CEACAM6	carcinoembryonic antigen-related cell adhesion molecule 6 (non-specific cross reacting antigen)	6.39±1.44
F5	coagulation factor V (proaccelerin, labile factor)	6.30±2.63
TCN1	transcobalamin I (vitamin B12 binding protein, R binder family)	6.28±1.69
FAM196B	family with sequence similarity 196, member B	6.27±0.59
CCL2	chemokine (C-C motif) ligand 2	6.18±1.16
LYZ	Lysozyme	6.14±0.87
KRT4	keratin 4	5.88±0.82
FAM111B	family with sequence similarity 111, member B, transcript variant 2	5.52±0.80
SLC17A4	solute carrier family 17, member 4, transcript variant 2	5.51±0.82
TAT	tyrosine aminotransferase	5.07±0.85

Down-regulated gene	Description	Fold change (vs. 2D)
EGR1	early growth response 1	0.032±0.008
SNAP25	synaptosomal-associated protein, 25kDa, transcript variant 1	0.068±0.006
DKK1	dickkopf WNT signaling pathway inhibitor 1	0.079±0.013
CYR61	cysteine-rich, angiogenic inducer, 61	0.156±0.026
FOS	FBJ murine osteosarcoma viral oncogene homolog	0.156±0.014
FGG	fibrinogen gamma chain (FGG), transcript variant gamma-A	0.160±0.012
GADD45B	growth arrest and DNA-damage-inducible, beta	0.170±0.030
JUNB	jun B proto-oncogene	0.177±0.025
OLFML2A	olfactomedin-like 2A, transcript variant 2	0.193±0.027
DUSP1	dual specificity phosphatase 1	0.201±0.021
QPRT	quinolinate phosphoribosyltransferase	0.216±0.028
PAPPA	pregnancy-associated plasma protein A, pappalysin 1	0.218±0.036
SOCS3	suppressor of cytokine signaling 3	0.228±0071
ST6GAL2	ST6 beta-galactosamide alpha-2,6-sialyltranferase 2, transcript variant 2	0.228±0.055
PAQR5	progestin and adipoQ receptor family member V, transcript variant 1	0.232±0.037
SULT2B1	sulfotransferase family, cytosolic, 2B, member 1, transcript variant 1	0.235±0.051
FOSB	FBJ murine osteosarcoma viral oncogene homolog B, transcript variant 2	0.237±0.018
SLC6A15	solute carrier family 6 (neutral amino acid transporter), member 15, transcript variant 3	0.240±0.008
LBP	lipopolysaccharide binding protein	0.242±0.003
HS3ST6	heparan sulfate (glucosamine) 3-O-sulfotransferase 6	0.247±0.060

The gene expression analysis was performed using total RNA of A549 cells cultured for 7 days in 2D or 3D (0.030% LA717). The DNA microarray assay was conducted with a GeneChip® Human Gene 2.0ST Array (Affimetrix, USA). Twenty most up- or

down-regulated protein-coding genes are shown. Data represent means ± SD of 3 independent experiments.

Supplementary Table S4. The IC_{50} value of anticancer drugs for A549 cells cultured in 2D and 3D with LA717 conditions

	IC_{50} (μM) (95% confidence interval) by ATP assay		IC_{50} (μM) (95% confidence interval) by imaging analysis
Conditions	2D	3D with LA717	3D with LA717
Paclitaxel	0.0027 (0.0024-0.0030)	0.0025 (0.0020-0.0029)	0.0027 (0.0007-0.0047)
Trametinib	0.0696 (0.0545-0.0847)	0.0204 (0.0143-0.0265)	0.0223 (0.0134-0.0312)
MK-2206	10.6642 (7.2690-14.0594)	0.7492 (0.5373-0.9611)	0.9950 (0.9380-1.0520)

ATP assay: A549 cells were inoculated at a density of 1,000 cells/well onto 96-well normal plates in DMEM (2D) and 96-well low attachment plates in DMEM (3D) with or without 0.030% (w/v) LA717. After 1 day of culture, anti-cancer drugs or DMSO (as vehicle control) were added to each well in triplicate. Following 7 days of culture, the numbers of live cells were counted using the ATP assay.

Imaging analysis: A549 cells were inoculated at a density of 1,000 cells/well onto 96-well low attachment plates in DMEM (3D) with 0.030% (w/v) LA717. After 1 day of culture, anti-cancer drugs or DMSO (as vehicle control) were added to each well in

triplicate. Following 7 days of culture, the numbers of A549 spheroids (diameter ≧ 50 μm) were counted using ArrayScan®VTI (ThermoFisherScientific, Massachusetts, USA).

The IC_{50} values were calculated with EXSAS by fitting a sigmoidal dose-response regression curve. Data represent means of 3 independent experiments.

Supplementary Table S5. The IC_{50} value of anti-cancer drugs for MCF-7 cells cultured in 2D and 3D with LA717 conditions.

	IC_{50} (μM) (95% confidence interval)			
	2D		3D with LA717	
Compounds	FBS(+)	FBS(-)	FBS(+)	FBS(-)
Paclitaxel	0.0020 (0.0018-0.0022)	0.0009 (0.0007-0.0011)	0.0021 (0.0019-0.0023)	0.0008 (0.0005-0.0011)
MK-2206	0.2098 (0.1776-0.2420)	0.2238 (0.1790-0.2687)	0.0741 (0.0571-0.0911)	0.4789 (0.2947-0.6630)

MCF-7 cells were inoculated at a density of 500 cells/well onto 96-well flat bottom cell culture plates in DMEM containing 10% FBS (2D, FBS(+)), 96-well collagen Ⅰ -coated plates in DMEM without FBS (2D, FBS(-)) or 96-well low attachment plates in DMEM with or without 10% FBS with 0.030% (w/v) LA717 (3D with LA717, FBS(+/-)). After 1 day of culture, anti-cancer drugs or DMSO (as vehicle control) were added to each well in triplicate. Following 7 days of culture, the numbers of live cells were counted using the ATP assay. The IC_{50} values were calculated with EXSAS by fitting a sigmoidal dose-response regression curve. Data represent means of 3 independent experiments.

Supplementary Figure S1. Scanning electron microscopy images of cell spheroid cultured in LA717-containing medium.

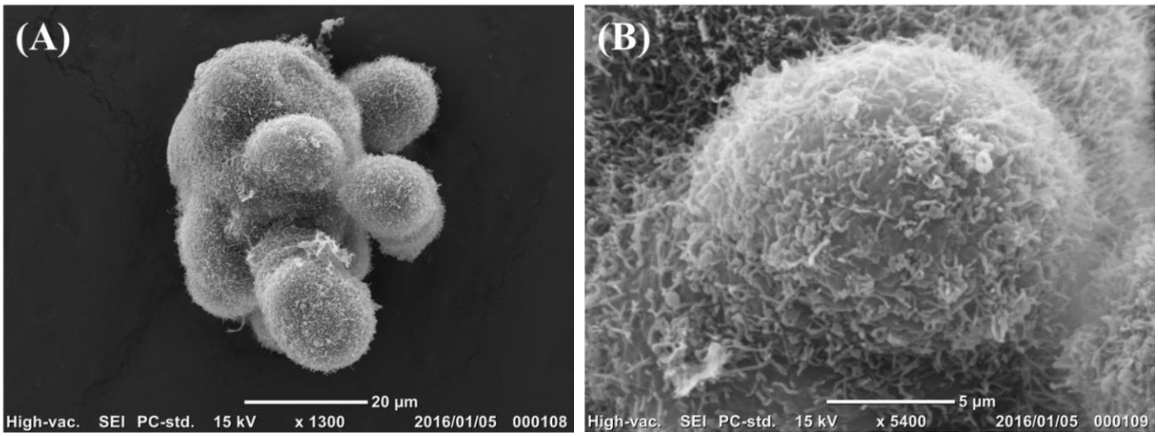

Samples were prepared from A549 cells cultured in the 3D condition on low-attachment cell culture plates (Corning Incorporated, #3474) in DMEM medium with LA717 for 7 days (Initial cell number; 1000 cells/100 μL/well). A549 cell spheroids were collected by centrifugation. The pellet was fixed with 2.5% glutaraldehyde in phosphate buffered saline (PBS), and rinsed three times with PBS. The fixed cell spheroids were dehydrated in 50%, 60%, 70%, 80%, 90%, 95% and 99.5% ethanol, with 5 minutes of incubation for each step. Such obtained spheroids were dried with t-butyl alcohol, sputter coated with platinum and then examined under the SEM, NeoScope™ JCM-6000 (JEOL Ltd., Tokyo, Japan). The bar represents 20 μm (A) and 5 μm (B).

Supplementary Figure S2. Single cells form clonal spheroids in LA717-containing medium.

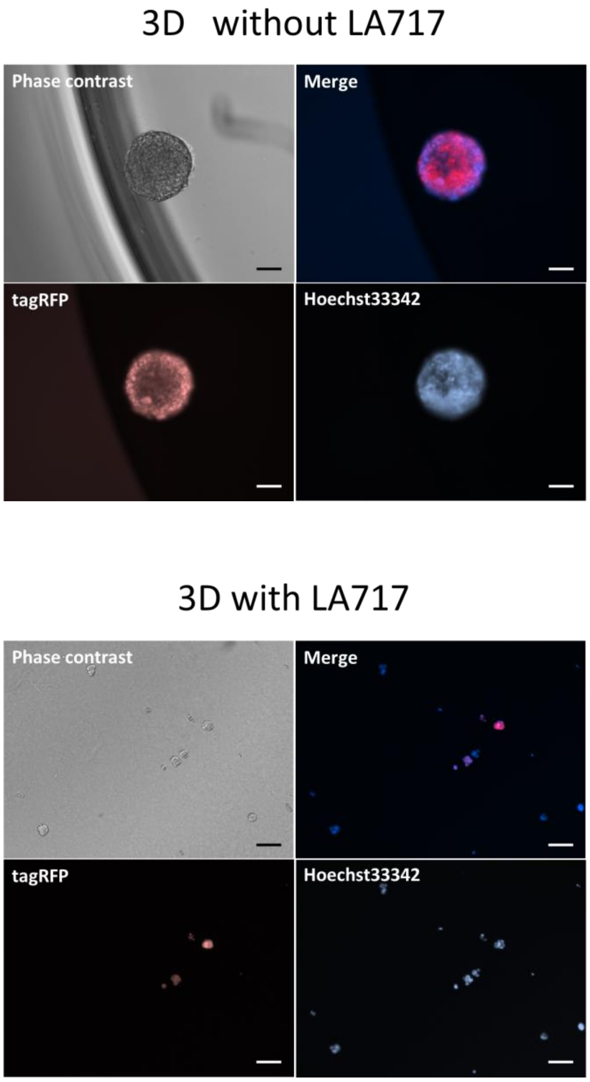

HEK-293 cells were obtained from American Type Culture Collection (Virginia, USA). Native HEK-293 cells and tagRFP expressing HEK-293 cells were dissociated to single cells and mixed at 1:1 ratio. Cells were seeded at a density of 2,000 cells/mL into DMEM without or with 0.03% (w/v) LA717, and were dispensed into the wells of a

96-well flat-bottom low-attachment plate at 100 μL/well (200 cells/well). After incubation for 4 days at 37°C, cells were observed by EVOS® FL Auto (ThermoFisherScientific, Massachusetts, USA).

In the absence of LA717, native HEK-293 cells and tagRFP expressing HEK-293 cells congregated at the edge of the well and formed large clumps, which expressed tagRFP in a mosaic pattern, demonstrating a mix of two populations. On the other hand, in the presence of LA717, HEK-293 cells formed small spheroids which consisted of either non-labelled or tagRFP-HEK293 cells. Only 3 mixed spheroids occurred out of 2,600 cells seeded (0.23%) on the low-attachment plates. The data suggest that the cells rarely form mixed spheroids in the medium containing LA717, but LA717 does not prevent cell to cell interaction and adhesion when dispensed cells accidentally attach to each other. Scale bars represent 200 μm. Shown images are representative of 3 independent experiments.

Supplementary Figure S3. Serum-free medium masks the high-sensitivity effect of 3D cultures to MK2206 on MCF7 cells.

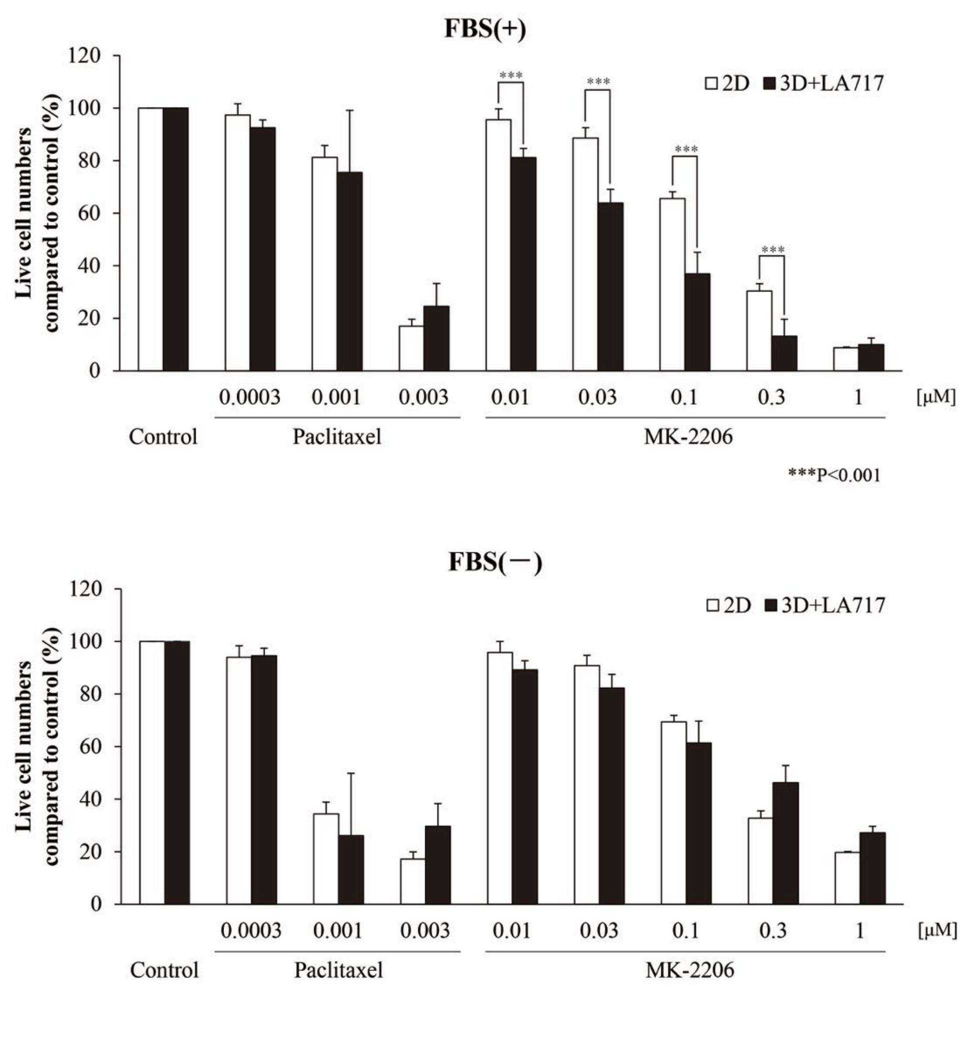

Anti-cancer drug-treated cells were prepared as described in Table S5. Following 7 days of incubation, the numbers of live cells were counted using the ATP assay. Data represent means ± SD of 3 independent experiments. Statistical significance was analyzed with Tukey's test.

Supplementary Figure S4. Effect of 3D culture methods on MK2206 sensitivity in A549 cells.

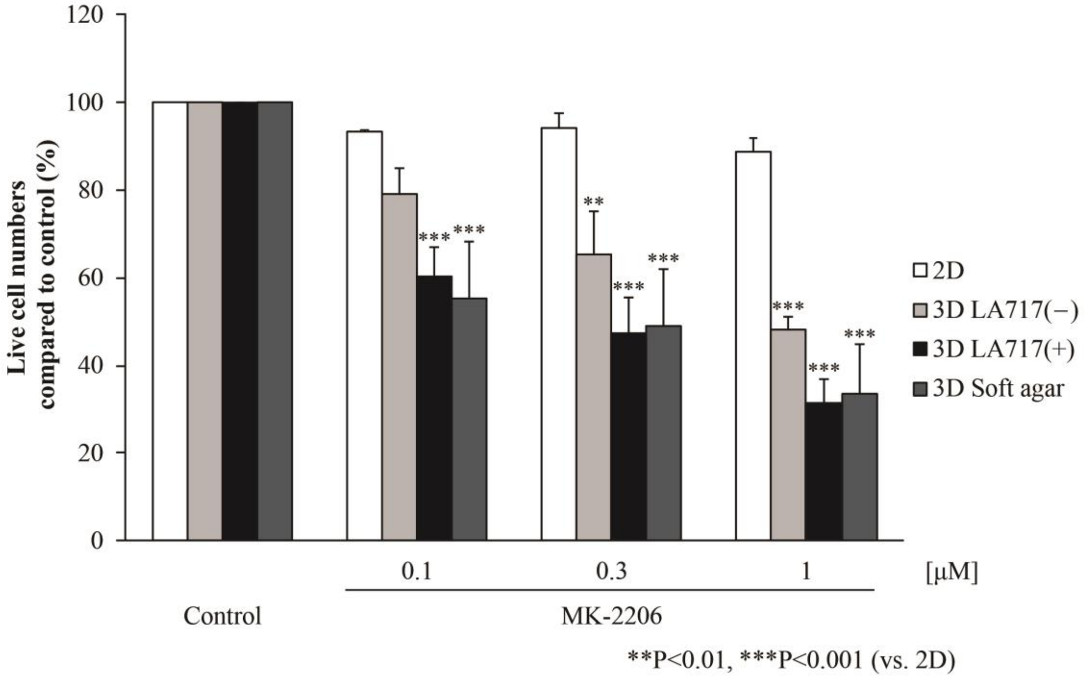

Sensitivity of A549 cells to MK-2206 was compared between 2D, 3D without LA717, 3D with LA717 and traditional 3D culture in soft agar (soft agar assay, described in Supplementary Materials & Methods). Following 7 days of incubation with MK-2206, the numbers of live cells were counted using ATP assay. Before the assay, 100 μL of PBS was added to each well and then mixed by pipetting, that digested agarose gel in the relevant well. 100 μL of cell suspension was transferred to white plates and used for ATP assay as described in Materials and Methods. Statistical significance was analysed by Tukey's test. Data represent means ± SD of 3 independent experiments.

Supplementary Figure S5. 3D culture with LA717 is suitable for HTS.

(a)

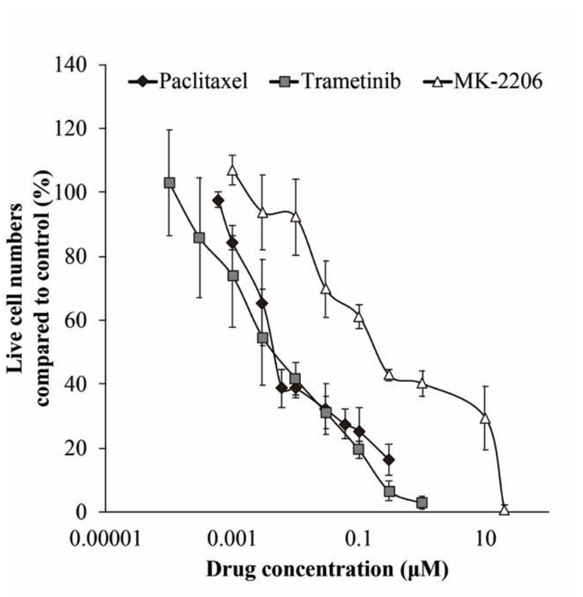

(b)

Compounds	IC_{50} (μM) (95% confidence interval)
Paclitaxel	0.0223 (0.0106-0.0340)
Trametinib	0.0147 (0.0082-0.0212)
MK-2206	0.6616 (0.3722-0.9509)

(a) A549 cells were inoculated at a density of 450 cells/well onto 384-well low attachment plates in DMEM with 0.030% (w/v) LA717. After 1 day of culture, anticancer drugs or DMSO (as vehicle control) were added to each well in triplicate.

Following 4 days of incubation, the numbers of live cells were counted using the ATP assay. Data represent means ± SD of 3 independent experiments. **(b)** The IC_{50} values were calculated with EXSAS by fitting a sigmoidal dose-response regression curve.

Supplementary Figure S6. 3D culture with LA717 is applicable to HCS.

(a)

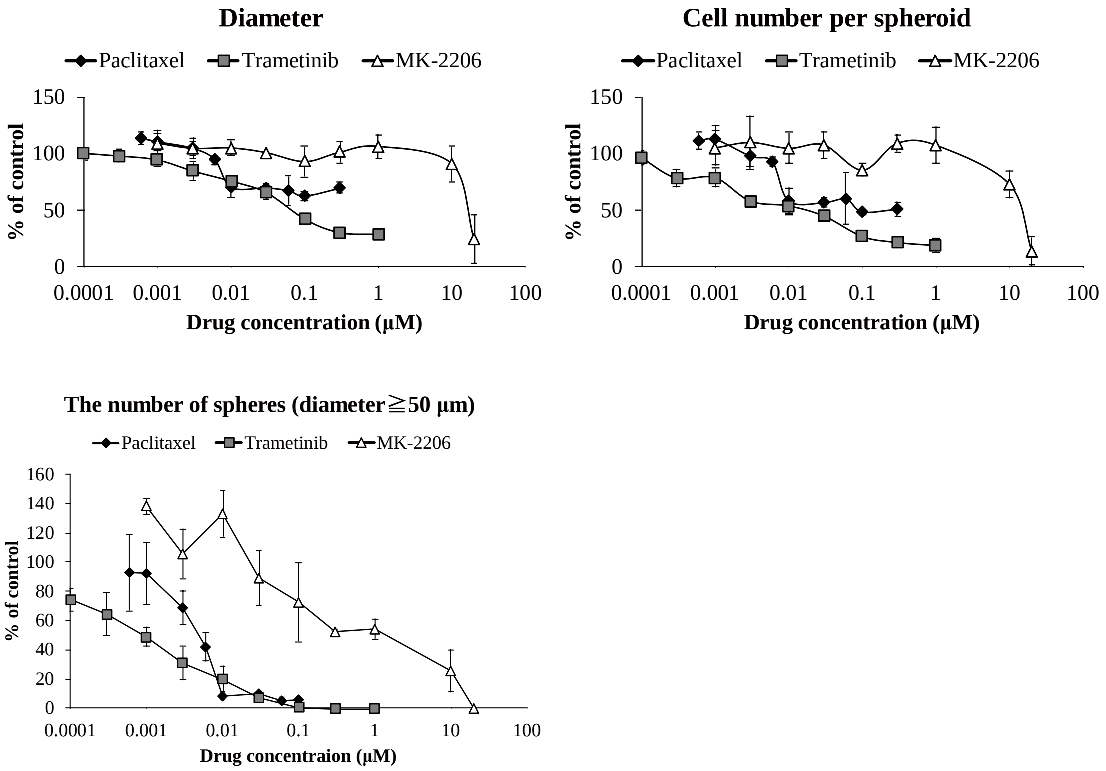

(b)

Compounds	IC_{50} (μM)<br>(95% confidence interval)
Paclitaxel	0.0052<br>(0.0042-0.0061)
Trametinib	0.0016<br>(0.0010-0.0022)
MK-2206	0.9977<br>(0.5722-1.4233)

A549 cells were inoculated at a density of 300 cells/well onto 384-well low attachment plates in DMEM with 0.030% (w/v) LA717. After 1 day of culture, anticancer drugs or DMSO (as vehicle control) were added to each cell culture well in triplicate. The cells were subsequently cultured for 7 days and the imaging analysis was performed with ArrayScan®VTI (ThermoFisherScientific, Massachusetts, USA). **(a)** The diameter of spheroids (left upper), the numbers of nuclei in spheroids (right upper), and the number of A549 spheroids (diameter ≧ 50μm) (lower). **(b)** The IC_{50} values were calculated with EXSAS by fitting a sigmoidal dose-response regression curve.

Supplementary Materials & Methods

Soft agar assay

Agarose (Nippon-Gene, Tokyo, Japan) was dissolved in water at 1.2% (w/v) by heating with a microwave oven and cool to 40°C in a water bath. For preparation of a base layer, equal volumes of 1.0% agarose solution and 2X DMEM prepared from powdered medium (Thermo Fisher Scientific, Massachusetts, USA) with 20% FBS were mixed to get DMEM containing 0.5% agarose and 10% FBS. It was then added at 50 μL/well on a 96well plate and set aside for 5 minutes to allow agarose to solidify. For preparation of a cell layer, 1.0% agarose solution, 2X DMEM with 20% FBS and A549 cell suspension were mixed, thus making the final concentration of agarose 0.35%. 75 μL of the cell suspension was placed on the base layer and set aside for 5 minutes. Culture medium containing DMSO or 3.5X concentration of MK-2206 was added at 50 μL/well. Cells were cultured at 37 °C in a humidified atmosphere with 5% CO_2.

Transfection

HEK-293 cells expressing tagRFP were generated as follows. The tagRFP/4T/O vector was constructed by inserting tagRFP sequence, which derived from pTagRFP-N vector (Evrogen, Moscow, Russia; #FP142), into the pcDNA™4T/O vector

(ThermoFisherScientific, Massachusetts, USA; #V102020). The tagRFP/4T/O vector was transfected into T-REx™-293 cells (ThermoFisherScientific; #R71007) using Nucleofector® 2b and Amaxa® Nucleofector® kit V according to manufacturer's protocol (Lonza, Basel, Switzerland). Stably transfected cells were selected in the culture medium containing 200 μg/mL of Zeocin (ThermoFisherScientific).

Time-lapse imaging (Supplementary video S1)

A549 cells were seeded at a density of 10,000 cells/mL into the indicated medium composition containing 0%(w/v) or 0.03% (w/v) of LA717, and was dispensed into the wells of a 96-well flat-bottom low-attachment plate at 100 μL/well. Time-lapse imaging was performed with EVOS® FL Auto (ThermoFisherScientific, Massachusetts, USA) at an interval of 30 minutes for 42 hours. Cells were cultured at 37 °C in a humidified atmosphere with 5% CO_2. The left panel shows the movement of A549 cells cultured in the medium without LA717, and the right panel shows those with LA717.
